# The effect of fatigue on plantar pressure distribution during running in view of running injuries

**DOI:** 10.1186/1757-1146-5-S1-P33

**Published:** 2012-04-10

**Authors:** Tine M Willems, Roel De Ridder, Philip Roosen

**Affiliations:** 1 Department of Rehabilitation Sciences and Physiotherapy, Ghent University, Ghent, Belgium, 9000

## Background

Several risk factors for the development of running injuries have been identified, however, the etiology is still not completely clear [1]. A number of prospective studies have identified gait-related risk factors for lower leg overuse injuries [2-6]. On the other hand, running injuries only develop by overloading the lower extremity. Fatigue can therefore be hypothesized to be a primary contributing factor. However, in determining injury etiology the relationship between the injury, the gait-related risk factors and overloading by fatigue is a complex model and the amount of contribution of each factor is difficult to assess. It might therefore be interesting to check 1) the interaction between fatigue and the roll-off pattern during running and 2)if fatigue generates specific gait-related risk factors for running injuries.

## Materials and methods

Prior to and after a 20 km run, force distribution underneath the feet of 52 participants was registered using Footscan® pressure plates while the participants ran shod at a constant self-selected pace. Peak force, mean force and impulse were registered underneath different zones of the foot. In addition, temporal data were derived and a medio-lateral force ratio was calculated during the roll-off.

## Results

**Figure 1:**
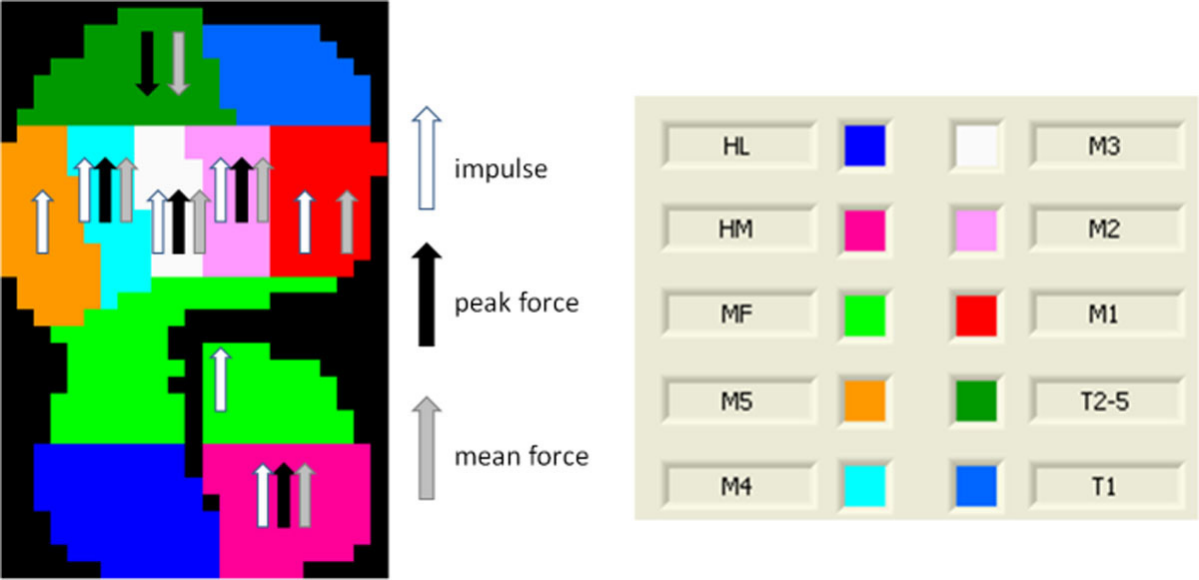
Significantly changed impulse, peak force and mean force for the different zones

After the run, increases in the loading of the forefoot, midfoot and medial heel were noted and decreases in loading of the lateral toes. Significant differences are presented in Figure 1. In the forefoot push off phase a more lateral pressure distribution was observed.

## Conclusions

Several of the significantly increased variables have been identified as risk factors for running injuries as stress fractures, patella-femoral pain syndrome and exercise-related lower leg pain. The results of this study demonstrated plantar pressure alterations after long-distance running which could give additional information related to several running injuries.
